# Light-Mediated Reduction in Photosynthesis in Closed Greenhouses Can Be Compensated for by CO_2_ Enrichment in Tomato Production

**DOI:** 10.3390/plants10122808

**Published:** 2021-12-18

**Authors:** Dennis Dannehl, Hans-Peter Kläring, Uwe Schmidt

**Affiliations:** 1 Faculty of Life Sciences, Albrecht Daniel Thaer-Institute of Agricultural and Horticultural Sciences, Division Biosystems Engineering, Humboldt-Universität zu Berlin, Albrecht-Thaer-Weg 3, 14195 Berlin, Germany; 2 Leibniz Institute of Vegetable and Ornamental Crops (IGZ) e.V., Theodor-Echtermeyer-Weg 1, 14979 Großbeeren, Germany; p.klaering@t-online.de

**Keywords:** closed greenhouse, CO_2_ concentration, photosynthetic photon flux density, photosynthesis, transpiration, water use efficiency, light intensity

## Abstract

Concepts of semi-closed greenhouses can be used to save energy, whereas their technical equipment often causes a decrease in the light received by the plants. Nevertheless, higher yields are achieved, which are presumably triggered by a higher CO_2_ concentration in the greenhouse and associated higher photosynthesis because of the technical cooling and the longer period of closed ventilation. Therefore, we examined the effects of photosynthetic photon flux density (PPFD) and CO_2_ concentration on plant photosynthesis and transpiration in tomato using a multiple cuvette gas exchange system. In a growth chamber experiment, we demonstrated that a light-mediated reduction in photosynthesis can be compensated or even overcompensated for by rising CO_2_ concentration. Increasing the CO_2_ concentration from 400 to 1000 µmol mol^−1^ within the PPFD range from 303 to 653 µmol m^−2^ s^−1^ resulted in an increase in net photosynthesis of 51%, a decrease in transpiration of 5 to 8%, and an increase in photosynthetic water use efficiency of 60%. Estimations showed that light reductions of 10% can be compensated for via increasing the CO_2_ concentration by about 100 µmol mol^−1^ and overcompensated for by about 40% if CO_2_ concentration is kept at 1000 instead of 400 µmol mol^−1^.

## 1. Introduction

Currently, finned tube heat exchangers are used in closed and semi-closed greenhouses for dehumidification and energy generation [1,2]. Even though these greenhouses contribute to sustainable production, the equipment in the roof area of such greenhouses provokes a light reduction of between 3 and 11%. It is well known that light reductions can decrease the productivity of plants. Kläring and Krumbein [3] and Marcelis et al. [4] observed 0.54% to 1.1% reductions in greenhouse tomato yields per 1% light reduction in Central and Northern Europe where shading is usually not necessary to avoid overheating of the plants. These plant responses are mainly attributed to a light-mediated reduction in photosynthesis. In this context, the fixation of the gaseous CO_2_ by ribulose-1,5-bisphospahte carboxylase/oxygenase (Rubisco) is the central step of the Calvin cycle, where this metabolic reaction provides carbon for growth [5]. The activity of Rubisco depends primarily on the partial pressure of the substrate CO_2_, and on temperature and photon flux [6]. Therefore, the mentioned light interception caused by the technical equipment in closed greenhouses is not acceptable for an efficient and sustainable greenhouse, unless the lack of light can be compensated for by improving other environmental conditions.

In contrast to open greenhouses, the climate in closed greenhouses on sunny days is characterized by high CO_2_ concentrations and relative humidity, where the temperature can be well controlled. Under these conditions, an increase in production of 10–34% was reported [7,8,9]. There is consensus in the scientific community that the higher CO_2_ concentration is responsible for the increased photosynthesis and associated yields. However, the wide range of reported results in such comparisons is caused by several factors such as the season, the cooling capacity of the closed greenhouse or the CO_2_ supply strategy of the open greenhouse [8,10]. Nonetheless, it is clear that Rubisco can bind oxygen and CO_2_. With a higher external CO_2_ concentration, the CO_2_ concentration in the mesophyll cells also increases, which means more CO_2_ is fixed and photorespiration is reduced [5].

In greenhouse experiments, the effects of light intensity and CO_2_ concentrations on crop photosynthesis have been studied. Nederhoff and Vegter [11] demonstrated that an increase in the CO_2_ concentration from 350 to 700 µmol mol^−1^ enhanced net photosynthesis of tomato plants by 27% at a photosynthetic photon flux density (PPFD) of 450 and 1350 µmol m^−2^ s^−1^ in the same manner. In cucumber and sweet pepper this increase was in the range from 18 to 53% depending on the season. Körner et al. [12] reported a crop gross photosynthesis increase of 50% in tomato and 55% in chrysanthemum if CO_2_ concentration increased from 400 to 1000 µmol mol^−1^. For free-air CO_2_ enrichment (FACE) experiments, Ainsworth and Rogers [13] presented in a meta-analysis that elevated CO_2_ concentration (567 µmol mol^−1^) stimulated light-saturated photosynthesis in C3 plants by an average of 31% compared to ambient CO_2_ concentration (366 µmol mol^−1^). However, the magnitude of the increase varied with the functional group and environment, from an average of 13% in crops to 46% in trees.

In conventional or semi-closed greenhouses, precise steady-state conditions are rarely maintained; environmental factors such as irradiance and CO_2_ concentrations may change rapidly. Therefore, a better understanding of the interacting effects of irradiance and CO_2_ concentrations on crop photosynthesis under variable environmental conditions is necessary. Previous research has focused on the phenomena underlying dynamic responses of photosynthesis to sunflecks and sunfleck utilization [14]. However, the effects of varying CO_2_ concentration with more than two CO_2_ concentration levels applied at different light intensities on dynamic photosynthesis are still unclear, although CO_2_ availability affects plant internal processes that limit the response of photosynthesis to fluctuating irradiance [15].

An advantage of closed compared to open greenhouses is also the reduction in water use. Usually, vapor pressure deficit (VPD) is lower in closed greenhouses and, therefore, transpiration is also lower. De Gelder et al. [16] observed a lower transpiration at high light intensities caused by a lower VPD in closed compared to open greenhouses. Another possible benefit is the recovery of water transpired by the plants by condensation in the cooling equipment. Dannehl et al. [7] reported that, in a tomato crop, 28% of the water supplied to the plants can be recycled in that manner. Plant transpiration is also sensitive to CO_2_ concentrations [17,18]. Whether this is also important in the context of plant production in closed greenhouses is still vague.

Therefore, the objective of the present study was to quantify the conditions under which a light-mediated reduction in photosynthesis can be compensated for by increased CO_2_ concentration. For this purpose, tomato plants were grown in a growth chamber. Crop photosynthesis and transpiration were measured using a multiple chamber gas exchange device under varying PPFD and CO_2_ concentrations. Finally, models were derived to quantify the effect of PPFD and CO_2_ concentration on photosynthesis and transpiration.

## 2. Results

### 2.1. Standard Values of Photosynthesis and Transpiration

Net photosynthesis on days two to four in the different cycles ranged from 8.88 to 17.09 µmol m^−2^ s^−1^ at 830 µmol m^−2^ s^−1^ PPFD, 8.34 to 15.89 µmol m^−2^ s^−1^ at 653 µmol m^−2^ s^−1^ PPFD, and 6.87 to 11.63 µmol m^−2^ s^−1^ at 453 µmol m^−2^ s^−1^ PPFD, respectively (Table 1). Three-way ANOVA resulted in significant effects of PPFD, the growth chambers, and the cycles (*p* < 0.001 for all three characteristics).

Similar to net photosynthesis, transpiration values ranged from 2.94 to 4.58 mmol m^−2^ s^−1^ at 830 µmol m^−2^ s^−1^ PPFD, 2.24 to 3.74 mmol m^−2^ s^−1^ at 653 µmol m^−2^ s^−1^ PPFD, and 2.00 to 3.03 mmol m^−2^ s^−1^ at 453 µmol m^−2^ s^−1^ PPFD, respectively (Table 2). Three-way ANOVA resulted in significant effects of PPFD, the growth chambers, and the cycles (*p* < 0.001 for all three characteristics).

### 2.2. Course of Photosynthesis and Transpiration Depending on PPFD

**Figure 1:**
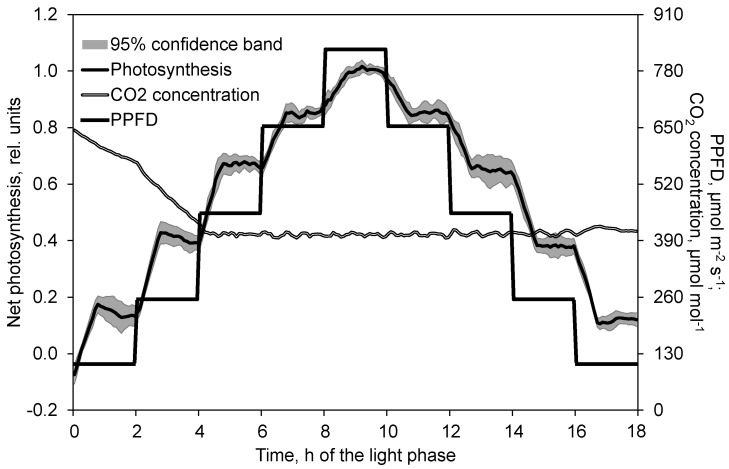
Effect of PPFD on net photosynthesis in the course of the light phase. Prior to analysis, photosynthesis data of each day and chamber are related to the average of the second hour at the maximum PPFD (time 9 to 10). Photosynthesis data show the mean and 95% confidence band of 8 replications. CO_2_ concentration data depict the corresponding mean of these replications.

The course of photosynthesis followed the course of the PPFD. As a consequence of the rapidly changed PPFD, net photosynthesis increased or decreased almost linearly. After 45 min it reached the new level and stayed at this level almost constantly (Figure 1).

**Figure 2:**
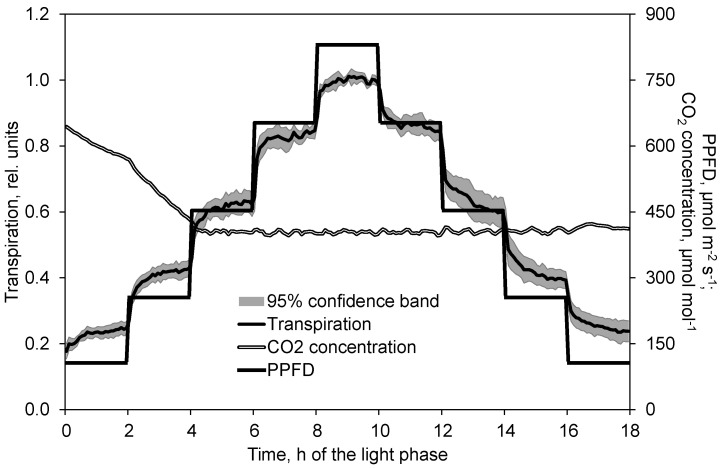
Effect of PPFD on transpiration in the course of the light phase. Prior to analysis, transpiration data of each day and chamber are related to the average of the second hour at the maximum PPFD (time 9 to 10). Transpiration data show the mean and 95% confidence band of 8 replications. CO_2_ concentration data depict the corresponding mean of these replications.

Transpiration rose or sloped steeply as the result of changed PPFD. Within a few minutes the gradient diminished continually. However, transpiration did not fully reach the steady state during the 2 h of constant PPFD (Figure 2).

### 2.3. Effect of Decreased PPFD Followed by Increasing CO_2_ Concentration on Photosynthesis and Transpiration

**Figure 3:**
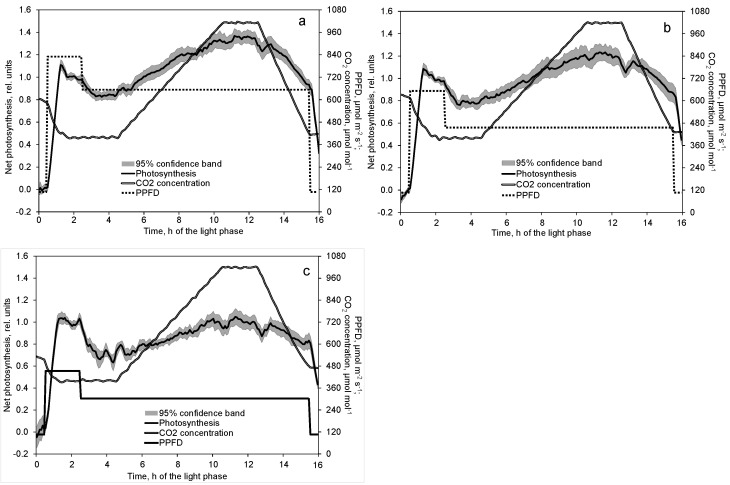
Effect of decreased PPFD followed by first increasing and later decreasing CO_2_ concentration on net photosynthesis at high (**a**), moderate (**b**), and low (**c**) basic light intensities. Prior to analysis, photosynthesis data of each day and chamber are related to the average of the second hour at increased PPFD (time 1.5 to 2.5). Photosynthesis data show the mean and 95% confidence band of 10 (**a**,**b**) and 9 (**c**) replications. CO_2_ concentration data depict the corresponding mean of these replications.

When PPFD suddenly increased from 106 µmol m^−2^ s^−1^ to the considerably higher values, net photosynthesis rose steeply for 45 min to a local maximum followed by a small decrement to a short steady state (Figure 3a–c). The local maximum was more distinct where the PPFD increase intervals were higher. After the decrease in PPFD to the lower level at a CO_2_ concentration of 400 µmol mol^−1^, photosynthesis decreased as already observed when measuring the light response (Figure 1). As photosynthesis data in Figure 3a–c are related to different standards, the relative decrement corresponds to the relative decrease in PPFD. In the further course of the days, photosynthesis rose with increasing CO_2_ concentration, stayed on a plateau at constant CO_2_ concentration, and dropped with decreasing CO_2_ concentration, respectively (Figure 3a–c). Due to the tightness of the chambers and the absence of a CO_2_ absorber, CO_2_ concentration in Figure 3b,c did not reach 400 µmol mol^−1^ by the end of the light phase because the plant’s photosynthesis was the only CO_2_ sink. This was also the case at the start of the light phase for reasons mentioned in Section 4.2. After 1.5 h of light, however, i.e., before the measurements for the standard started, the CO_2_ concentration always reached the 400 µmol mol^−1^ level.

**Figure 4:**
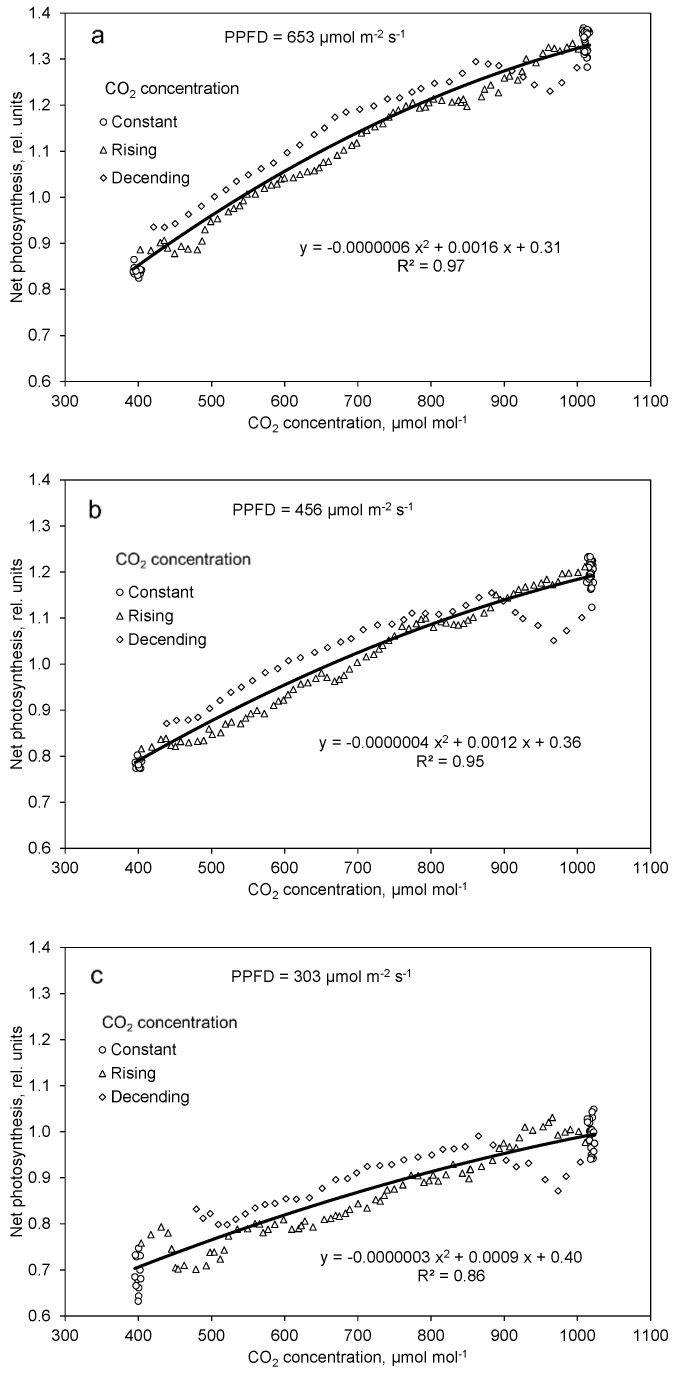
Effect of constant, increasing, and decreasing CO_2_ concentration on net photosynthesis at high (**a**), moderate (**b**), and low (**c**) light intensities. Data are means of 10 (**a**,**b**) and 9 (**c**) replications. Included are all data after one hour of adaptation to the corresponding PPFD (time 3.5 to 15.5).

From Figure 3a–c follows a significant correlation of net photosynthesis and CO_2_ concentration, which is displayed in Figure 4a–c. The gradient of this function increases with rising PPFD. Only a very small saturation effect in photosynthesis with increasing CO_2_ concentration could be observed in the investigated range from 400 to 1000 µmol mol^−1^ for all examined PPFD levels. At the same CO_2_ concentration, apparent photosynthesis is greater at dropping than rising CO_2_ concentration (Figure 4a–c).

**Figure 5:**
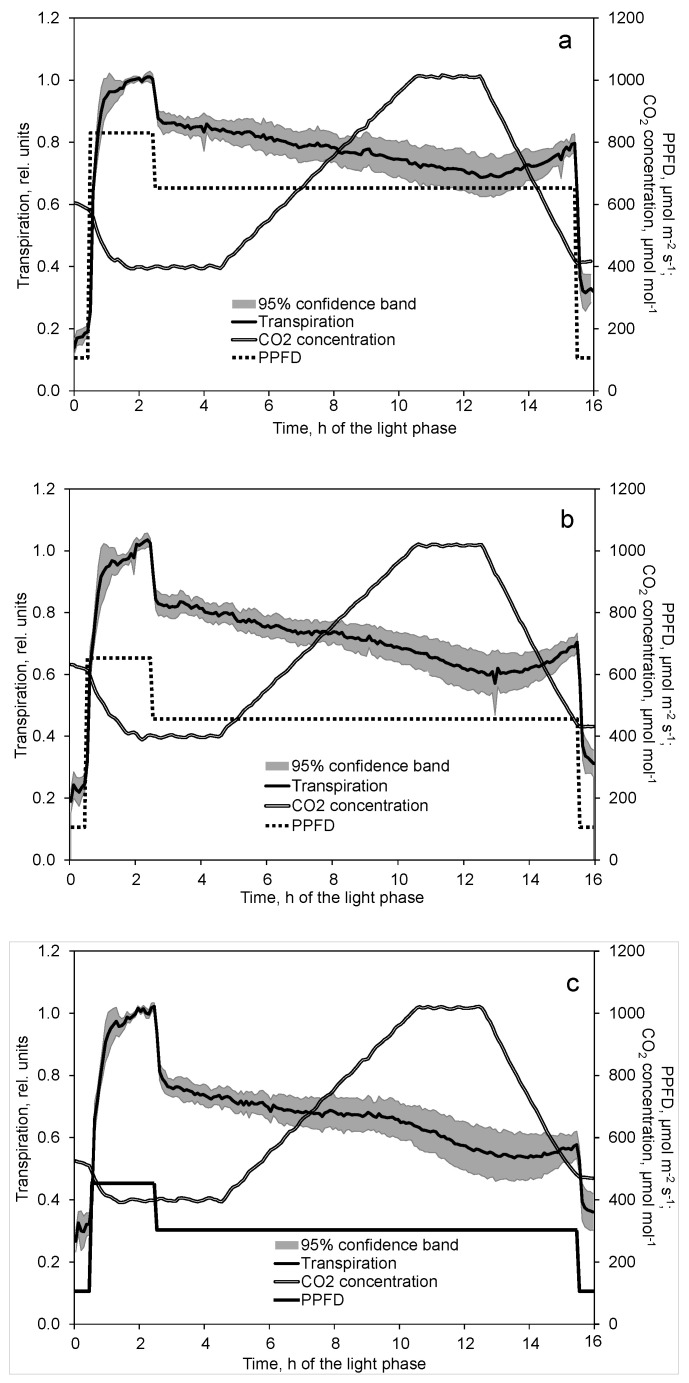
Effect of decreased PPFD followed by first increasing and later decreasing CO_2_ concentration on transpiration at high (**a**), moderate (**b**), and low (**c**) basic light intensities. Prior to analysis, transpiration data of each day and chamber are related to the average of the second hour at increased PPFD (time 1.5 to 2.5). Transpiration data show the mean and 95% confidence band of 10 (**a**,**b**) and 9 (**c**) replications. CO_2_ concentration data depict the corresponding mean of these replications.

Transpiration rose steeply for about 30 min following the sudden high PPFD increase (Figure 5a–c). The gradient was even higher in transpiration than in photosynthesis. However, neither a local peak nor a steady state was observed.

Transpiration continued to slightly increase as already observed in the light response measurements (Figure 2). This also concerned the effect of decreasing the PPFD. After a rapid drop, transpiration in the further course slightly diminished with increasing CO_2_ concentration but increased again with decreasing CO_2_ concentration (Figure 5a–c). Fractions of 30%, 54% and 71% of the variance in transpiration could be explained by the variation in CO_2_ concentration at low, moderate, and high light PPFD, respectively.

**Figure 6:**
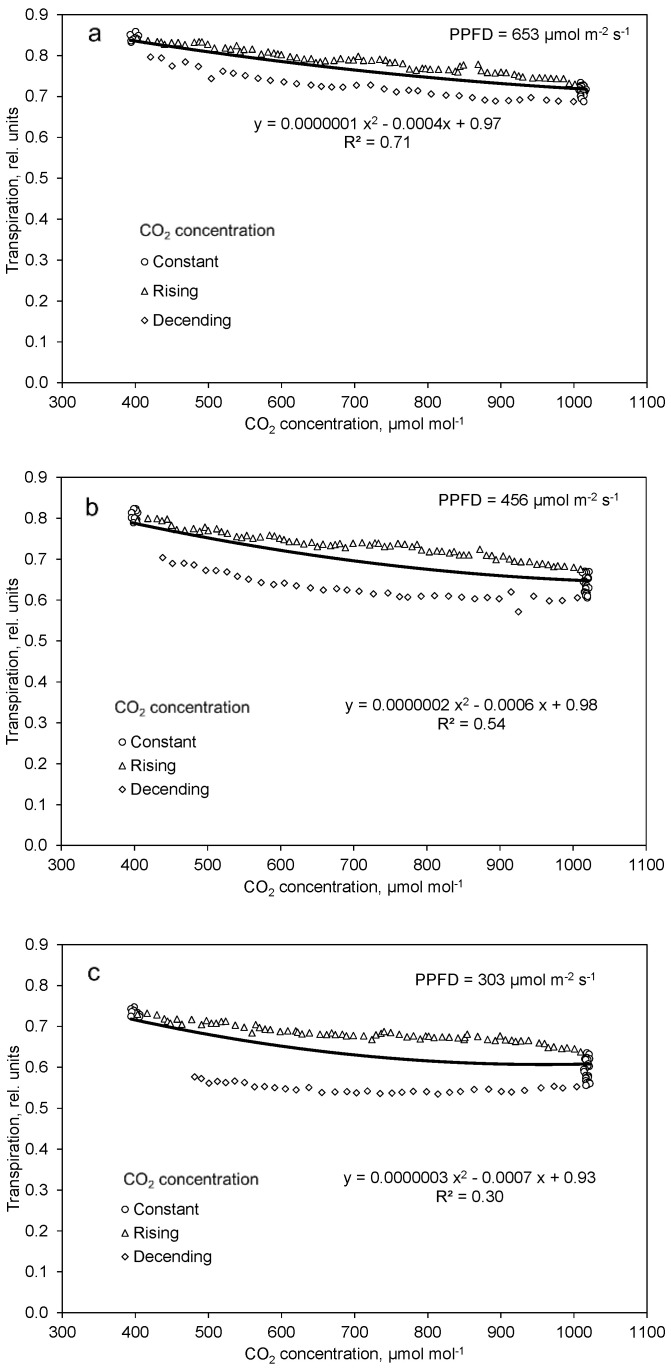
Effect of constant, increasing and decreasing CO_2_ concentration on transpiration at high (**a**), moderate (**b**), and low (**c**) light intensities. Data are means of 10 (**a**,**b**) and 9 (**c**) replications. Included are all data after one hour of adaptation to the corresponding PPFD (time 3.5 to 15.5).

Transpiration was higher during increasing CO_2_ concentration than decreasing CO_2_ concentration (Figure 6a–c). Interestingly, this difference was greatest at the lowest PPFD.

### 2.4. Photosynthesis and Transpiration Affected by PPFD and CO_2_ Concentration

PPFD (PPFD, µmol m^−2^ s^−1^), CO_2_ concentration (CO_2_, µmol mol^−1^) and their interaction clearly affected net photosynthesis (P*_net_*, rel. units):

(1) $$P_{\mathrm{net}}=2.86\cdot \left(1-e^{0.0890-0.00142\cdot \mathrm{PPFD}}\right)\cdot \left(1-e^{-0.169-0.00149\cdot {\mathrm{CO}}_{2}}\right),\;R^{2}=0.99$$

**Figure 7:**
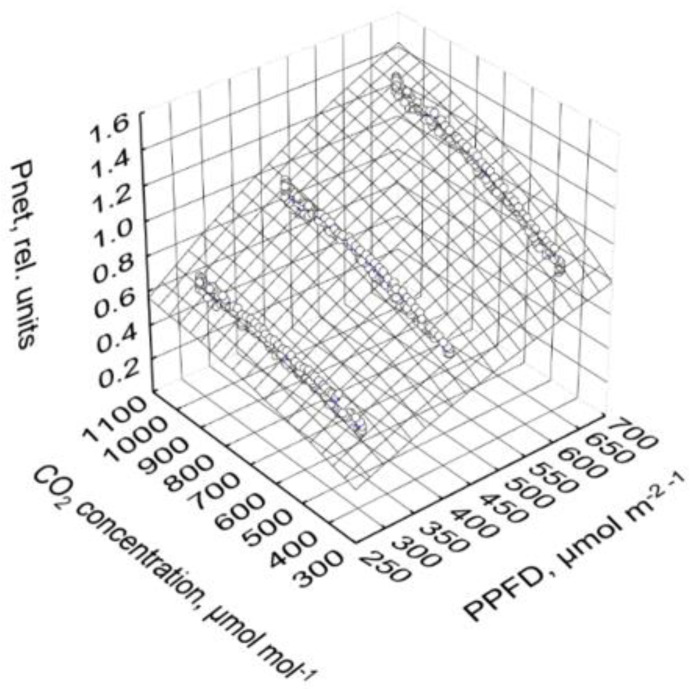
Net photosynthesis depending on PPFD and CO_2_ concentration. Prior to analysis, data in each cycle and chamber are related to the corresponding average of the second hour at the highest PPFD of 830 µmol m^−2^ s^−1^ of the third day (high PPFD, time 1.5 to 2.5) of the corresponding cycle and chamber. Points depict the mean values of all cycles and both chambers after adaptation to the corresponding PPFD (time 3.5 to 15.5), and the area is defined by the non-linear regression function: $P_{\mathrm{net}}=2.86\cdot \left(1-e^{0.0890-0.00142\cdot \mathrm{PPFD}}\right)\cdot \left(1-e^{-0.169-0.00149\cdot {\mathrm{CO}}_{2}}\right)$.

In the observed ranges from 303 to 653 µmol m^−2^ s^−1^ PPFD and 400 to 1000 µmol mol^−1^ CO_2_ concentration, only relatively weak saturation effects of photosynthesis were observed (Figure 7). Based on Equation (1), net photosynthesis increased by 51% when the CO_2_ concentration was increased from 400 to 1000 µmol mol^−1^ in all PPFD treatments.

Transpiration (TR, rel. units) was markedly affected by PPFD but only slightly influenced by CO_2_ concentration:

(2) $$\;\mathrm{TR}=12.4\cdot \left(1-e^{-0.0129-0.0000921\cdot \mathrm{PPFD}}\right)-0.000149\cdot {\mathrm{CO}}_{2},{\;R}^{2}=0.96$$

**Figure 8:**
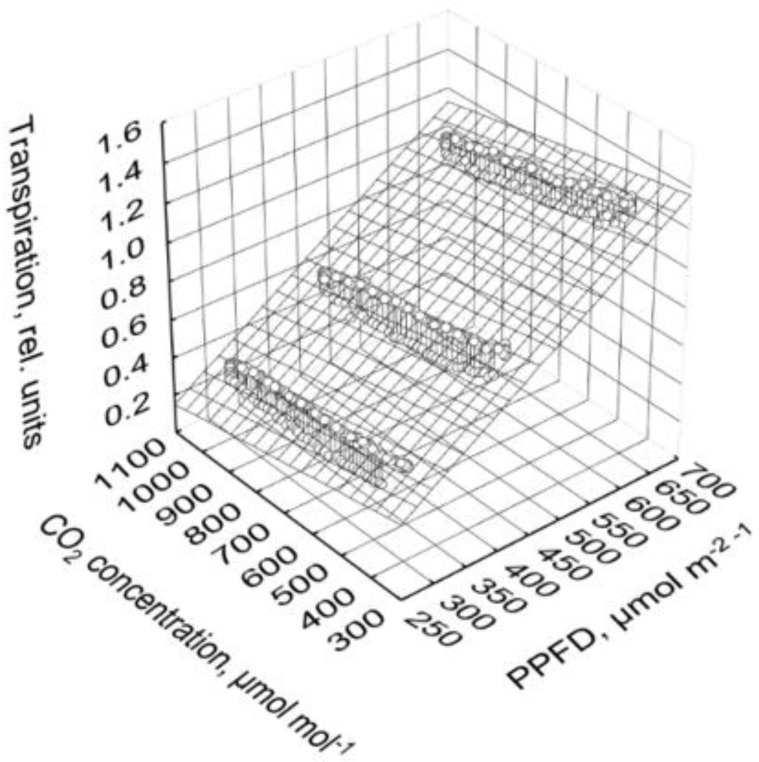
Transpiration depending on PPFD and CO_2_ concentration. Prior to analysis, data in each cycle and chamber are related to the corresponding average of the second hour at the highest PPFD of 830 µmol m^−2^ s^−1^ (high PPFD, time 1.5 to 2.5) of the third day of the corresponding cycle and chamber. Points depict the mean values of all cycles and both chambers, and the area is defined by the non-linear regression function:$\;\mathrm{TR}=12.4\cdot \left(1-e^{-0.0129-0.0000921\cdot \mathrm{PPFD}}\right)-0.000149\cdot {\mathrm{CO}}_{2}$.

No interaction of PPFD and CO_2_ concentration or a saturation with increasing PPFD was observed (Figure 8).

The differences between the values during increasing and decreasing CO_2_ concentration (Figure 6a–c and Figure 8) could be related to the hour of the light phase (t, h of the light phase):

(3) $$\mathrm{TR}=11.2\cdot \left(1-e^{-0.0176-0.000103\cdot \mathrm{PPFD}}\right)-0.0000678\cdot {\mathrm{CO}}_{2}-0.00938\cdot t,\;R^{2}=0.99$$

Based on Equation (3), transpiration decreased by 5%, 7%, and 8% when the CO_2_ concentration was increased from 400 to 1000 µmol mol^−1^ at 653, 456, and 303 µmol m^−2^ s^−1^ PPFD, respectively.

WUE of photosynthesis (WUE, (mmol CO_2_) (mol H_2_O)^−1^)) was significantly affected by CO_2_ concentration:

(4) $$\mathrm{WUE}=0.614-0.000100\cdot \mathrm{PPFD}+0.00136\cdot {\mathrm{CO}}_{2},{\;R}^{2}=0.76$$

**Figure 9:**
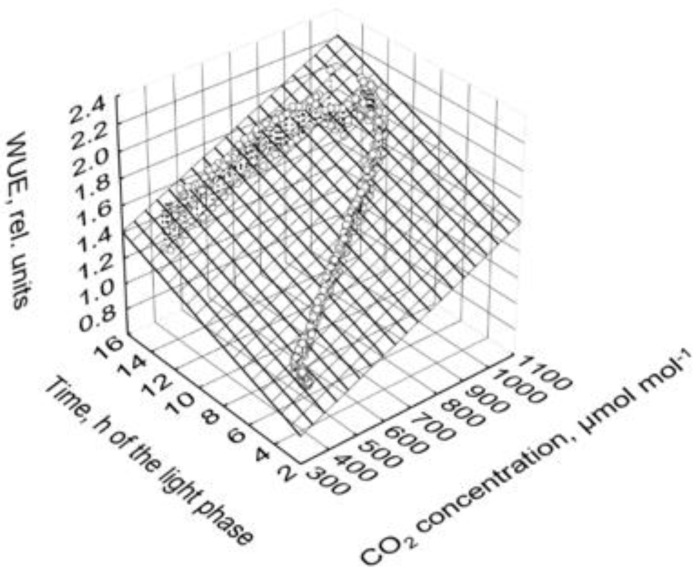
WUE depending on PPFD and CO_2_ concentration. Prior to analysis, data in each cycle and chamber are related to the corresponding average of the second hour at the highest PPFD of 830 µmol m^−2^ s^−1^ (high PPFD, time 1.5 to 2.5) of the third day of the corresponding cycle and chamber. Points depict the mean values of all cycles and both chambers, and the area is defined by the linear regression function: $\mathrm{WUE}=0.423+0.00101\cdot {\mathrm{CO}}_{2}+0.0423\cdot t$.

Photosynthesis and transpiration increased with increasing PPFD in a similar manner (Figure 2 and Figure 3). Therefore, the effects of PPFD on WUE were not significant in the observed range from 303 to 654 µmol m^−2^ s^−1^ and can be neglected (Equation (4)). However, the decrease in the transpiration in the course of the light phase (Equation (3)) resulted in a significant increase in WUE (Figure 9):

(5) $$\mathrm{WUE}=0.423+0.00101\cdot {\mathrm{CO}}_{2}+0.0423\cdot t,\;R^{2}=0.91$$

Based on Equation (5), WUE increased by 60% when the CO_2_ concentration was increased from 400 to 1000 µmol mol^−1^ in all PPFD treatments.

## 3. Discussion

Measuring photosynthesis and transpiration depending on CO_2_ concentration is an experimentally challenging goal. There are intricate problems related to the gradients from the ambient to the intercellular CO_2_ and H_2_O concentrations. These gradients significantly depend on the air movement around the leaf. Therefore, systems controlling the environment inside a cuvette are less situated for the measurement of the effects of the CO_2_ concentration on photosynthesis and transpiration of complete canopies because they intensively mix the air close to the leaf’s surface and thus affect the difference between the ambient CO_2_ and H_2_O concentration and that inside the leaf. Therefore, CO_2_ response curves measured in those systems are mostly related to the intercellular CO_2_ concentration, e.g., [19].

In the present experiment, photosynthesis and transpiration was measured using the commercial equipment of BERMONIS (Steinbeis GmbH & Co. KG; Stuttgart, Germany). This system has many leaf cuvettes in different positions and avoids the destruction of the leaf boundary layer. Air movement within the cuvettes is laminar and the velocity is about 4.6 cm s^−1^. These low air velocities can be expected within a dense canopy in greenhouse when ventilation is closed [20].

Another challenge is the measurement under changing environmental conditions. To the best of our knowledge, measurements on the effect of the ambient CO_2_ concentration on photosynthesis in whole plant systems have only been carried out in the steady state conditions and usually include two or three CO_2_ concentration levels [11,12]. These systems usually measure the inlet and outlet CO_2_ concentration with the same sensor resulting in a time lag between taking the two measurements, in the present case of 2.5 min. Therefore, if ambient CO_2_ concentration was changing, data of inlet CO_2_ concentration were interpolated in order to fit it to the corresponding CO_2_ concentration taken from the cuvettes. A similar correction was proposed by Stinziano et al. [19] for measuring the response at linearly increasing or decreasing CO_2_ concentrations in a LI-6800 Portable Photosynthesis System equipped with the Multiphase Flash Fluorometer and Chamber (LI-COR Inc., Lincoln, NE, USA).

In the present study, a time delay of photosynthesis and transpiration was observed with changing environmental conditions. Rapidly changed radiation results in slowly altering leaf temperature and stomatal conductance. The adaptation of the photosynthesis to these changed conditions may implicate a significant time delay [15]. In the present experiment, leaf temperature increased by about 5 K when PPFD increased from 106 to 830 µmol m^−2^ s^−1^, and stomatal conductance increased by a factor of 3.5 (data not shown). In addition, it is well known that stomata need some time to reach the maximum degree of opening, especially at the beginning of the day, whereby the maximum of photosynthesis and transpiration at defined light conditions is delayed. More important, however, is the function of the enzyme Rubisco, which plays a very important role in the fixation of CO_2_ [6]. The transition from the inactive to the active state of Rubisco can take some time, which can also explain our observed delay [5].

The increases of both net photosynthesis and transpiration with increasing PPFD (Figure 1 and Figure 2) are in agreement with literature data on tomato [12,21,22]. However, the observed response to changed PPFD was much faster in transpiration than in photosynthesis. Although transpiration is largely regulated by the opening of the stomata, photosynthesis is not only subject to stomatal opening but also to the activation of Rubisco, the key enzyme in the Calvin cycle, to fix CO_2_ [6]. These processes need time and may be one explanation for the delayed photosynthesis compared to the transpiration. In these complex processes, the difference in diffusion velocity between CO_2_ and H_2_O in the intercellular air spaces and in the mesophyll cells must also be taken into account [23]. In the air, the resistance is greater for CO_2_ than for H_2_O, which is based on different diffusion coefficients of these parameters (H_2_O = 2.6 × 10^−5^ m^2^ s^−1^ at 25 °C and 1 bar; CO_2_ = 1.5 × 10^−5^ m^2^ s^−1^).

Increasing CO_2_ concentration in the range of 400 to 1000 µmol mol^−1^ compensated and partly overcompensated for a decrease in PPFD by about 170 µmol m^−2^ s^−1^, which was a 27%, 43%, and 49% decrease in the high, moderate, and low PPFD day course, respectively (Figure 4a–c). The effect of high CO_2_ concentration on net photosynthesis was greater at high than at low PPFD because of the multiplicative interaction of PPFD and CO_2_ concentration, as shown in Equation (1) and Figure 7. Similar results were also found by Leakey et al. [24] and Tomimatsu et al. [25]. The 51% increase in net photosynthesis (Equation (1)) with increasing CO_2_ concentration from 400 to 1000 µmol mol^−1^ is in good agreement with data by Körner et al. (2009). Apparent net photosynthesis was higher at decreasing than increasing CO_2_ concentration (Figure 4a–c), which can be interpreted as a delay in the response of the intercellular CO_2_ concentration to the changed ambient conditions. We mapped the net photosynthesis data onto the CO_2_ concentration of 15 min (a and b) and 20 min (c) before it harmonized the effects of the CO_2_ concentration in the upward and downward courses. The coefficient of determination increased from 0.97 to 0.98 (a), 0.95 to 0.96 (b), and 0.86 to 0.90 (c), and the regression coefficients were negligibly affected (data not shown). It is most likely that the relatively slow response of the stomata was the main reason for this time lag.

Interestingly, the time lag in photosynthesis after the change in PPFD was greater than that after the change in CO_2_ concentration. One reason may be that PPFD considerably affected transpiration and stomatal conductance concurrently, whereas these quantities are only marginally influenced by CO_2_ concentration (Figure 2 and Figure 5a–c; stomatal conductance not shown). To the best of our knowledge, data describing a possible time delay due to the leaf boundary layer resistance in the response of photosynthesis and transpiration to changed environmental conditions are not available for dense plant canopies. This is difficult because it requires measurements or good estimations of the very low wind speed in such canopies. As in small leaf cuvettes, and most gas exchange chambers and greenhouses, the air is well mixed, which is a necessary preposition for the control of the environment by cooling, heating, CO_2_ supply, or dehumidification [26,27]. However, mixing the air generates turbulences and destroys the natural boundary layer and results in a very fast adaptation of measured photosynthesis to changed environmental conditions. Teitel et al. [28] measured photosynthesis and transpiration of a sweet pepper crop in a greenhouse (18 m × 24 m). Air was supplied via pads on the one side and was exhausted on the other side, resulting in directed movement at low velocity within the canopy. Although photosynthesis responded to rapidly changed intensity of solar radiation within 3 to 4 min, the maximum photosynthesis was measured with a delay of 30 min to the maximum solar radiation [28]. This is similar to the observed response to rapidly increasing and decreasing PPFD (Figure 1). A similar large delay in the photosynthesis response to rapidly changed PPFD was also observed in a greenhouse system for real-time gas exchange measurements [21]. Switching artificial illumination on or off required much more time for photosynthesis adaptation in the measurements than in the time course obtained by a sophisticated photosynthesis model [29].

In contrast to photosynthesis, transpiration decreased with increasing CO_2_ concentration (Figure 5a–c). According to Equation (3), transpiration decreased by 5% to 8% when CO_2_ concentration increased from 400 to 1000 µmol mol^−1^, where the highest value was obtained at the lowest PPFD. A reduced transpiration at higher CO_2_ concentration was also reported by other authors for tomato plants [30]. Moreover, unlike photosynthesis, apparent transpiration was higher at rising than dropping CO_2_ concentration (Figure 6a–c). The main reason for this difference was the decrease in transpiration in the course of the day. This was also reported under field conditions by Bunce [17].

Based on the behavior of photosynthesis and transpiration, and according to Equation (5), WUE increased by 60% when CO_2_ concentration increased from 400 to 1000 µmol mol^−1^. These plant responses can contribute to lower freshwater consumption in crop production in closed greenhouses. An effect of PPFD on WUE could not be observed in the range from 303 to 653 µmol m^−2^ s^−1^ (Figure 9). Of course, if PPFD draws near the light compensation point of net photosynthesis then WUE converges to zero.

**Figure 10:**
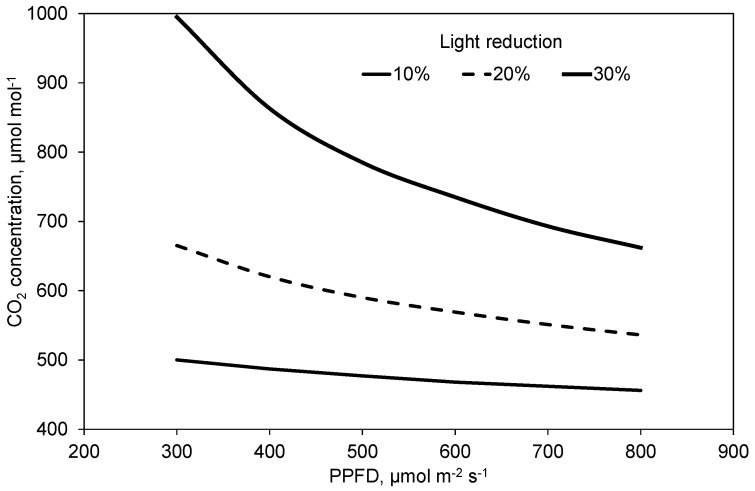
Compensation lines for light reduction. Curves depict the CO_2_ concentration necessary to reach the equal net photosynthesis as obtained without light reduction at 400 µmol mol^−1^ CO_2_ concentration. Lines are estimated based on Equation (1).

Due to the reduction in fossil resources for heating, closed greenhouses are designed to provide a significant incremental yield increase. In a review, De Gelder et al. (2012) specify this increment to be 10% to 20%. They relate it mainly to the increased CO_2_ concentration in semi-closed greenhouses. However, such greenhouses usually include more expensive equipment. Sometimes this equipment reduces the light transmission of the greenhouse cover [1,31], potentially reducing the plant’s photosynthesis and yield. However, technical cooling and dehumidification of the greenhouse air may keep the greenhouse windows closed under increasing solar radiation and outside temperature [8]. Then, the reduction in light transmission can be compensated for by continued CO_2_ supply to the greenhouse air as demonstrated in Figure 3a–c. Figure 10 depicts that the compensation point does not depend on the reduction in PPFD only, but also on its initial level due to the interaction of both variables in Equation (1).

**Figure 11:**
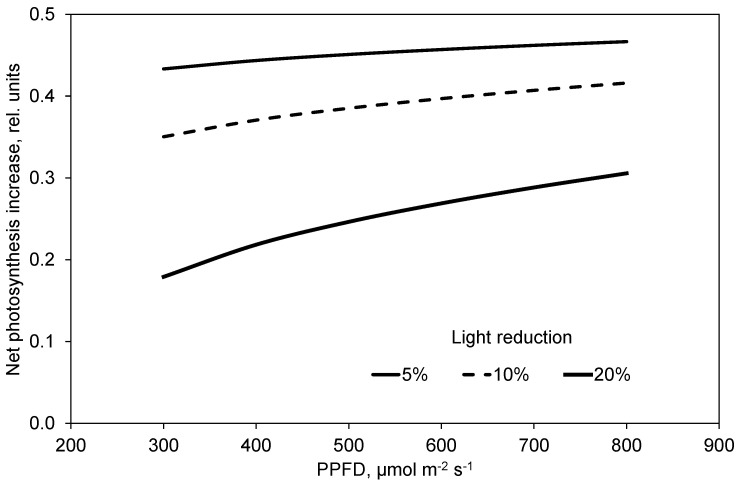
Net photosynthesis increment in a closed greenhouse at a CO_2_ concentration of 1000 µmol mol^−1^ compared to a non-closed greenhouse without light reduction at 400 µmol mol^−1^.

Figure 11 depicts the net photosynthesis increment that is obtained at a CO_2_ concentration of 1000 µmol mol^−1^ compared to the photosynthesis without light reduction at 400 µmol mol^−1^. It means, for example, that plants in a closed greenhouse with a 10% lower light transmission may have a 40% higher photosynthesis compared to plants in an open greenhouse where the CO_2_ concentration is much lower. This is the case under conditions of high solar radiation or high outside temperature. At lower temperature and radiation, conventional greenhouses are not ventilated, and may also maintain high inside CO_2_ concentrations, and therefore have a benefit compared to closed greenhouses with lower light transmission. The time under such conditions in the course of a vegetation period under Northern conditions is likely greater than the phases when technical cooling is applied in the closed greenhouse. However, the potential photosynthesis is much greater when solar radiation requires cooling in order to keep the greenhouse closed and CO_2_ concentration is high (Figure 7). Thus, the theoretical comparison of both types of greenhouses over a complete season is not trivial due to the difficult estimation of the ventilation opening behavior and the CO_2_ supply strategies of the non-closed greenhouse, in addition to the equipment of the semi-closed greenhouse. Therefore, experiments are of high value. Quian et al. [10] reported that the yield in a semi-closed greenhouse with 150 and 350 W m^−2^ cooling capacity was 6% and 10% higher than in an open greenhouse. Heuvelink et al. [32] simulated a yield increase for a closed greenhouse of 17%, which was in good agreement with our measurements. CO_2_ concentration in the closed greenhouse was kept at 1000 µmol mol^−1^, whereas it dropped to 450 µmol mol^−1^ in the conventional greenhouse at high temperature and solar radiation. Dannehl et al. (2013) reported a yield increment in tomato by 32% in a closed greenhouse with a 11% lower light transmission compared to an otherwise identical conventional greenhouse. The CO_2_ target concentration in this closed greenhouse was 800 µmol mol^−1^. This CO_2_ concentration was maintained for a longer period in a closed greenhouse, which again increased the yields. Here, it has to be considered whether the costs of higher energy consumption in a closed greenhouse are covered by higher yields. In this context, the exact relationship between CO_2_ fixation in the crop and yield development should be investigated in further trials in order to optimize the control of microclimatic conditions in greenhouses as a function of online photosynthesis measurements.

## 4. Materials and Methods

### 4.1. Plant Material

Investigations were conducted on tomato plants (*Solanum lycopersicum* L. cv. Pannovy; Novartis International AG, Basel, Switzerland) from 20th March until 30th of June. Seeds were sown on 20th March in gravel and germinated in a growth chamber at 27 °C. Subsequently, seedlings were transplanted to 1 L pots filled with gravel and grown in a greenhouse (52° 21′ N, 13° 18′ E) at temperature set points for heating during night and day of 18 and 20 °C, respectively. Ventilation was opened automatically at 24 °C. Plants were irrigated daily with a nutrient solution which was prepared according to recommendations for hydroponic production of De Kreij et al. [33]. The plants were trained in accordance with horticultural practice: all side shoots and leaves below the trusses with red fruits were removed. Pollination was facilitated by vibrating flowering trusses twice a week. Sixty-one days after sowing, 12 plants were transferred into two growth chambers (Yorck, Mannheim, Germany) with a ground area of 10 m^2^ each. The gravel was rinsed from the roots and the plants were then hung by their shoots on a wire. The roots were set in 21 L polyethylene containers with 15 L constantly aerated nutrient solution. The nutrient solution taken up by the plants was periodically replenished. The containers were covered with black-and-white plastic foil (white side facing the outside) to inhibit algae growth and evaporation. In the chambers, the plants were arranged in two rows of six plants. They were illuminated at a PPFD of 500 µmol m^−2^ s^−1^ generated by high-pressure sodium discharge lamps AGRO SON-T 400 W (Philips, Eindhoven, The Netherlands) for a 16 h photoperiod. The air temperature, relative humidity, and CO_2_ concentration at day and night were adjusted to 25 °C, 70%, and 400 µmol mol^−1^, respectively. During the dark phase, the CO_2_ concentration increased to about 500 to 600 µmol mol^−1^ due to the plant’s natural respiration, the higher CO_2_ concentration in the growth chamber building, the presence of people in the chamber to take care of the plants and the equipment, and the absence of a CO_2_ absorber. After a growing period of 22 days in the chambers, climatic treatments and measurements of the photosynthesis and transpiration started.

### 4.2. Experimental Design

Five measurement cycles were run in the two growth chambers. In addition to temperature and relative humidity, the CO_2_ concentration in the chambers could be controlled by supplying technical pure CO_2_ to the air. Furthermore, PPFD in the chambers could be varied. The lamps could be switched in five independent groups realizing a PPFD of 106, 149, 176, 197, and 201 µmol m^−2^ s^−1^ on the top of the plants. Combining these groups allowed the installation of a wide range of illumination.

Before starting a new cycle, all cuvettes were reassigned to new leaves, in order to respond to the changing architecture of the growing plants and to avoid any long-term effect the cuvettes could have on the leaves.

During the first day of each cycle, except for the last cycle, the light course increased step-wise in two-hour increments from 106 to 255, 453, 653, and finally to peak intensity at 830 µmol m^−2^ s^−1^ before decreasing via the same steps to 106 µmol m^−2^ s^−1^. The CO_2_ concentration was maintained at 400 µmol mol^−1^ during the entire day. The light phase comprised 18 h. In the last cycle, these measurements were omitted. Therefore, the first four cycles included four days whereas the fifth cycle consisted of only three days.

**Figure 12:**
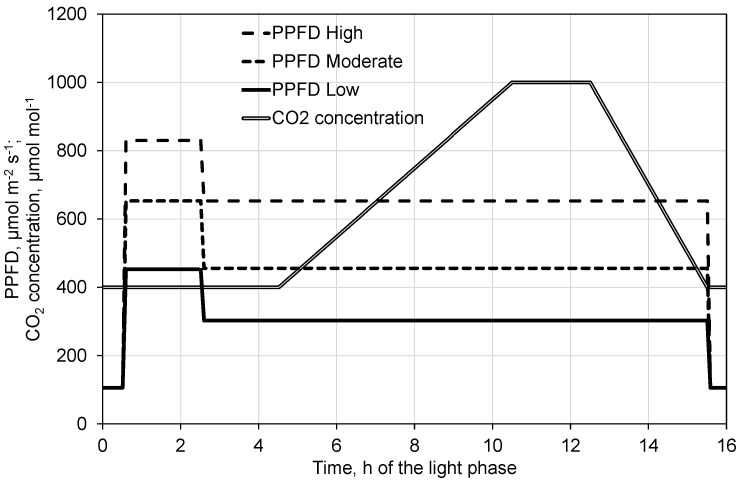
Set points for PPFD and CO_2_ concentration control applied in order to assess the compensation effect for decreased PPFD by increasing CO_2_ concentration.

During the following three days of each cycle, both the PPFD and the CO_2_ concentration were varied. The day phase always started with 30 min at a PPFD of 106 µmol m^−2^ s^−1^ in order to adapt the plants to the light after the dark phase and finished with 30 min at the same light intensity. At each of the following three days, the compensation was assessed for decreased PPFD by increasing CO_2_ concentration. This was performed at three different basic light intensities (‘low’, ‘high’, and ‘moderate’, in this sequence). After the first 30 min at 106 µmol m^−2^ s^−1^, the PPFD was increased for 2 h to 453, 830, and 653 µmol m^−2^ s^−1^ and subsequently decreased and maintained at 303, 653, and 456 µmol m^−2^ s^−1^ for the low, high, and moderate light treatments, respectively (Figure 12). After another 2 h of adaptation of the plants to the decreased PPFD, the CO_2_ concentration in the growth chambers was increased linearly over 6 h from 400 to 1000 µmol mol^−1^, maintained at this level for 2 h before being decreased to 400 µmol mol^−1^ over 3 h (Figure 12). Subsequently, the PPFD in the chambers was adjusted for 30 min at 106 µmol m^−2^ s^−1^, resulting in a total duration of the light phase on these days of 16 h (Figure 12).

The night phase in the chambers was used for all necessary controls and handling of the plants and the equipment. The respiration of the plants and the presence of people in the growth chambers resulted in an increase in the CO_2_ concentration due to the absence of a CO_2_ absorber.

An extremely low value of 8.88 µmol m^−2^ s^−1^ on day 3 (high PPFD) of cycle 4 in chamber A was the result of changes of the position of leaves above the cuvettes. Therefore, some cuvettes were reinstalled on the next day, which was not the rule within a cycle. There was an error in the CO_2_ control of chamber A on day 2 of the first cycle (low PPFD). Therefore, the data were not included in the data analysis.

### 4.3. Measurements of Photosynthesis and Transpiration

**Figure 13:**
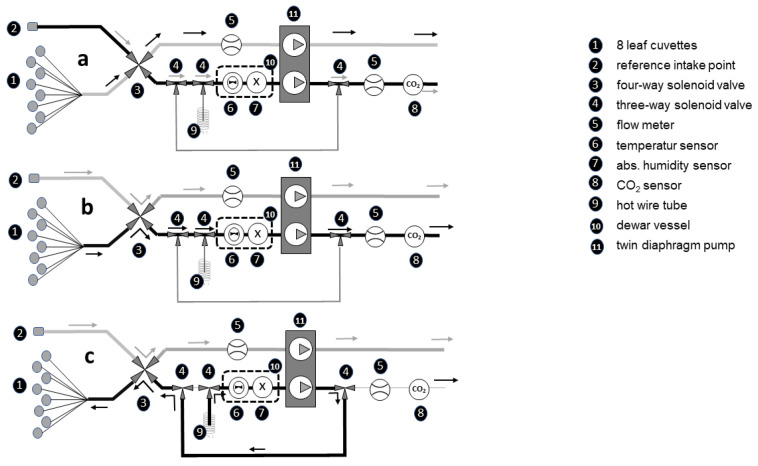
Pneumatic diagram of BERMONIS system with reference measuring pathway (**a**), cuvette measuring pathway (**b**), and back drying pathway (**c**).

In both growth chambers, the BERMONIS leaf cuvette-based gas exchange system (Steinbeis GmbH & Co. KG; Stuttgart, Germany) was used to measure net photosynthesis and transpiration of tomato plants. The analysis in the BERMONIS system is based on an open gas exchange measurement. Eight leaf cuvettes are attached accordingly to 8 different leaves on several plants. A total air flow of 133 m^3^ h^−1^ is drawn through the 8 cuvettes. Using an air flow of 16.6 m^3^ h^−1^ per cuvette and an average flow cross-section of 10 cm^2^ resulted in an air flow velocity in the 8 cuvettes of 4.6 cm s^−1^ on average. With this low velocity and the arrangement of the inflow opening and the suction hose at maximum distance from the leaf surface, the aim is to maintain the natural boundary layer conditions in the leaf. The extracted air from the 8 cuvettes is mixed and fed into a dewar vessel to measure the absolute humidity and the CO_2_ concentration from the measuring chamber (Figure 13a. Reference air from the surroundings of the cuvette measuring points is drawn in cyclically every 5 min and the absolute humidity and CO_2_ content are determined (Figure 13b). The mean value of leaf transpiration and CO_2_ uptake of the 8 measuring points is calculated from the difference in absolute humidity and CO_2_ content between the cuvette air and the reference air. At higher humidity, the system cyclically switches on a dehumidification routine to dry the tubes and cuvettes in order to prevent condensation (Figure 13c).

In the present experiment, eight fully developed leaves on four plants in the middle of both growth chambers were measured. Four cuvettes were fixed on leaves in the upper part of the canopy in a nearly horizontal position and exposed to the direct irradiance of the light fixtures. Another four cuvettes were placed in the lower ranges. The net photosynthesis and plant transpiration rates were measured every 5 min, which were expressed as µmol m^−2^ s ^−1^ and mmol m^−2^ s^−1^, respectively. Photosynthetic water use efficiency (WUE) was calculated as the ratio of net photosynthesis and transpiration.

### 4.4. Data Analysis

In order to use the data of the five cycles and the two chambers as 10 replications in the analysis, the cuvettes were always fixed on leaves of the same development stage and in similar positions. Nevertheless, the light absorption, and possibly also the activities of the leaf parts embedded in the cuvettes, were not exactly equal. Plants may age and adapt to the conditions in the growth chamber over the time of the experiment in different ways. The conditions in the two growth chambers were likely not exactly identical. In order to exclude these effects, the data were standardized prior to the analyses. For the days of each cycle with varying CO_2_ concentration, the mean values of photosynthesis and transpiration of the last hour before decreasing the higher initial radiation from each light treatment (low, high, and moderate light intensity, Figure 12, time 1.5–2.5) were used as the standards. All data of this day were divided by the corresponding standard. This was analogously done with the data of the first day of each cycle representing the light response curves. Here, the mean value of the last hour at 830 µmol m^−2^ s^−1^ PPFD (time 9–10) was used as the standard.

In addition, nonlinear regression models for photosynthesis, transpiration, and WUE depending on PPFD and CO_2_ concentration were developed based on data of the CO_2_ response measurements. In order to avoid effects of inertness of photosynthesis and transpiration after sudden changes of the PPFD, only data after one hour of these changes were included; that is, from one hour after decreasing the initial high PPFD until PPFD was decreased to 106 µmol m^−2^ s^−1^ close to the end of the light phase (Figure 12, time 3.5 to 15.5). Here, the average of the second hour at the initial high PPFD of the high PPFD treatment (day 3, time 1.5 to 2.5 at 830 µmol m^−2^ s^−1^ PPFD and 400 µmol mol^−1^ CO_2_ concentration) was used as the standard for the corresponding cycle and chamber.

Original data of photosynthesis and transpiration is presented as means with the 95% confidence intervals. The effects of PPFD and CO_2_ concentration on photosynthesis, transpiration, and WUE are illustrated in multiple partly non-linear regression models. Model parameters were estimated by Gauss–Newton iteration using the software Statistica (Version 6.1, Statsoft Inc., Tulsa, OK, USA).

## 5. Conclusions

In closed greenhouses, the additional equipment may reduce the transmission of light to the plants. However, the reduction in photosynthesis can be compensated or even overcompensated for by keeping the CO_2_ concentration high at high solar radiation and outside temperature. This effect depends mainly on the cooling capacity and associated ventilation behavior. In the present experiment, with a high leaf boundary layer resistance for water vapor and CO_2_, a saturation of photosynthesis was not reached at a CO_2_ concentration of 1000 µmol mol^−1^. An increase in the CO_2_ concentration above this threshold in dense greenhouses may further improve canopy photosynthesis and production and should be investigated. As transpiration slightly decreased with raising CO_2_ concentration, WUE consequently significantly increased.

## Figures and Tables

**Table 1:** Average values of photosynthesis during the second hour of the maximum PPFD of the cycles and chambers at CO_2_ concentration of 400 µmol mol^−1^.

Cycle/Chamber	Net Photosynthesis, µmol m^−2^ s^−1^		
	At High PPFD 830 µmol m^−2^ s^−1^	At Moderate PPFD 653 µmol m^−2^ s^−1^	At Low PPFD 453 µmol m^−2^ s^−1^
1/A	17.09	15.89	11.63
1/B	15.58	14.83	10.62
2/A	15.07	12.43	10.57
2/B	14.43	11.26	9.01
3/A	13.81	11.48	7.78
3/B	11.91	9.31	7.79
4/A	8.88	10.58	7.55
4/B	10.02	8.06	6.92
5/A	13.13	11.35	8.63
5/B	9.63	8.34	6.87

Columns depict data for the days when the light response curve and the effect of compensation for decreasing PPFD by increasing CO_2_ concentration at high, moderate, and low PPFD levels were measured.

**Table 2:** Average values of transpiration during the second hour of the maximum PPFD of the cycles and chambers at a CO_2_ concentration of 400 µmol mol^−1^.

Cycle/Chamber	Transpiration, mmol m^−2^ s^−1^		
	At High PPFD 830 µmol m^−^^2^ s^−1^	At Moderate PPFD 653 µmol m^−^^2^ s^−1^	At Low PPFD 453 µmol m^−^^2^ s^−1^
1/A	4.58	3.74	3.03
1/B	4.33	3.55	2.95
2/A	4.11	3.25	2.63
2/B	4.00	3.18	2.38
3/A	3.81	2.74	2.15
3/B	3.23	2.24	2.00
4/A	2.94	2.99	2.01
4/B	3.26	2.59	2.02
5/A	3.95	3.13	2.19
5/B	3.41	2.56	2.02

Columns depict data for the days when the light response curve and the effect of compensation for decreasing PPFD by increasing CO_2_ concentration at high, moderate, and low PPFD levels were measured.

## Data Availability

Data is contained within the article.
